# How Egg Case Proteins Can Protect Cuttlefish Offspring?

**DOI:** 10.1371/journal.pone.0132836

**Published:** 2015-07-13

**Authors:** Valérie Cornet, Joël Henry, Didier Goux, Emilie Duval, Benoit Bernay, Gildas Le Corguillé, Erwan Corre, Céline Zatylny-Gaudin

**Affiliations:** 1 Université de Caen Basse-Normandie, F-14032, Caen, France; 2 UMR 7208, BOREA CNRS INEE, F-14032, Caen, France; 3 Plateforme Post-génomique PROTEOGEN, F-14032, Caen, France; 4 CMAbio, F-14032, Caen, France; 5 Plateforme ABiMS, Station biologique de Roscoff (UPMC-CNRS), F-29688, Roscoff, France; Laboratoire de Biologie du Développement de Villefranche-sur-Mer, FRANCE

## Abstract

*Sepia officinalis* egg protection is ensured by a complex capsule produced by the female accessory genital glands and the ink bag. Our study is focused on the proteins constituting the main egg case. *De novo* transcriptomes from female genital glands provided essential databases for protein identification. A proteomic approach in SDS-PAGE coupled with MS unveiled a new egg case protein family: SepECPs, for *Sepia officinalis* Egg Case Proteins. N-glycosylation was demonstrated by PAS staining SDS-PAGE gels. These glycoproteins are mainly produced in the main nidamental glands. SepECPs share high sequence homology, especially in the signal peptide and the three cysteine-rich domains. SepECPs have a high number of cysteines, with conserved motifs involved in 3D-structure. SDS-PAGE showed that SepECPs could form dimers; this result was confirmed by TEM observations, which also revealed a protein network. This network is similar to the capsule network, and it associates these structural proteins with polysaccharides, melanin and bacteria to form a tight mesh. Its hardness and elasticity provide physical protection to the embryo. In addition, SepECPs also have bacteriostatic antimicrobial activity on GRAM- bacteria. By observing the SepECP / *Vibrio aestuarianus* complex in SEM, we demonstrated the ability of these proteins to agglomerate bacteria and thus inhibit their growth. These original proteins identified from the outer egg case ensure the survival of the species by providing physical and chemical protection to the embryos released in the environment without any maternal protection.

## Introduction

Oviparous species lay eggs that ensure the sustainability of the species. Laid eggs contain energy reserves and a protective structure to allow embryonic development. In direct contact with the environment, these eggs are exposed to mechanical stress, microbial infection and predation. The egg capsule or eggshell, secreted by the genital tract in sauropsida [1–3], specific glands like the silk gland in spiders [4,5] or nidamental glands in sharks [6,7], represent the first natural barrier between the developing embryo and the environment. The proteins present in egg cases or eggshells play a dual role in physical and chemical protection. Egg proteins have been largely described in avian eggshells. The hen eggshell is composed of a wide range of proteins with a structural role, a role in immune defense, or both [8]. Thus, physical protection is provided by common structural proteins like keratin-like proteins [9] and collagen [10,11] that form the fibers of eggshell membranes, and by other eggshell matrix-specific proteins: ovocleidin [12,13] and ovocalyxin [14,15]. Some eggshell matrix proteins like ovocalyxin-36 provide a dual protection by exhibiting antimicrobial activity [16,17]. Finally, immune defense proteins like β-N-acetylglucosaminidase, lysozyme and ovotransferrin are associated to eggshell proteins [18]. In marine oviparous animals, the main studies of egg case proteins have been carried out on shark and gastropod eggs. In dogfish eggs, the hard matrix structure composed of collagen-like proteins gives strength and resilience to the egg-case, thereby an effective mechanical protection [19]. This protective structure allows exchanges with the environment and has antifouling properties that could be provided by collagen proteins [20]. Unlike the dogfish egg-case, the marine gastropod egg capsule possesses elastic properties given by structural proteins similar to keratin to face the huge hydrodynamic forces generated by water velocity and shocks [21]. This egg capsule avoids bacterial infection in gastropods in their early stages of development [22,23]. Eggshell antifouling activity was evidenced in several species, e.g. in gastropods [24]. In *Aplysia* eggs, physical and chemical protection is provided by two specific types of proteins. Capsulin, a membrane-associated protein produced by the albumen gland is involved in mechanical protection [25], while the glycoproteins Aplysianin A and E ensure immune defense with bacteriostatic activity on GRAM-/+ bacteria [26,27]. Moreover, egg masses from invertebrates [28,29] can provide chemical defense against bacteria in addition to the physical barrier in early-stage embryos and during embryonic development.

In cephalopods, egg encapsulation is also essential for embryo development [30]. Diversity of egg morphology, size and structure among octopus, nautilus, squid and cuttlefish species has largely been studied [30–32]. Egg structure modifications among these different species respond to adaptations to environmental and developmental constraints [33]. In *Sepia officinalis*, the egg capsule is formed from secretions of the female genital apparatus [30,34]. The mature oocyte passes through the oviductal gland and is covered by a first secretion forming the first inner layer of the egg. Then, the oocyte is released inside the mantle cavity and embedded into the nidamental glands and the ink bag secretions to form the outer layer. Finally, the embedded oocyte is fertilized by spermatozoa in the buccal mass and hung to egg-laying substrate. Although egg capsule morphology is well described [30–32], very few proteins or peptides have been identified from cephalopod eggs. Only a few pheromones have been identified so far. In squid, a β-microseminoprotein is produced in the reproductive gland and embedded in the outer tunic of the egg capsule [35]. This contact pheromone has an extremely aggressive effect on males in the context of sexual selection. So far, *Sepia officinalis* egg mass studies have only revealed a waterborne pheromone (ILME) involved in oocyte transport during egg-laying [36], and a sperm-attracting peptide (SepSAP) that facilitates fertilization [37].

This study is aimed at identifying the egg case proteins of the cuttlefish *Sepia officinalis* involved in embryo protection, using a proteomic approach. We also performed histological studies to monitor egg case matrix structure and its development. Finally, to elucidate the mechanisms involved in chemical protection, we assayed antimicrobial activity from eggs and egg-case proteins.

## Material and Methods

### Animal collection

All cuttlefish were caught in the Baie de Seine (Channel Sea, 49°19'18.24"N—0°20'42.26"O) between January and June. They were maintained in 1 000-liter outflow tanks at 16°C with a natural photoperiod at the Marine Station of Luc/Mer (49°19’5.869”- 0°21’3.348”) (University of Caen, France,). The rearing structures were adapted to improve the welfare of animals. No specific permits were required for the described field studies and the common cuttlefish is not endangered or protected species.

### Animal research

All experiments have been carried out in accordance with relevant guidelines and regulations regarding the care and the use of animals for the experimental procedures. The protocol was approved by the local Ethical Committee on Animal Research “Comité d'Ethique Normandie en Matière d'Expérimentation Animale” (C2EA-54). Experiments should be carried out in accordance with the European Communities Council Directive of 24 November 1986 (86/609/EEC) regarding the care and use of animals for experimental procedures. All efforts were made to minimize suffering. Mature cuttlefish of both sexes were anesthetized with ethanol 3%. After exsanguination, the genital apparatus and the central nervous system (CNS) were dissected, frozen in liquid nitrogen and stored at −80°C until RNA isolation and protein extraction.

### Egg Collection

“Couples” formed by one male for two females were put in several tanks for them to reproduce, and egg-laying initiation was overseen. Eggs from different females were separately collected directly after spawning and also after 15 days’ incubation at 16°C for histological studies.

### Optical Microscopy

Eggs were fixed for 72 h at 4°C in Davidson solution (10% filtered seawater, 30% alcohol at 95%, 20% formaldehyde at 40%). After dehydration in successive baths of ethanol at 70, 95, and 100%, the eggs were included into paraffin wax. Sections of 7 μm were cut and stained with Prenant Gabe trichromatic [38], or Periodic Acid Schiff (PAS). Slides were acquired on a Scan scope CS (Leica biosystems) and treated with Aperio Image Scope v12.1.0.5029 software. Egg capsule (including the outer and inner layers of the extrachorion and the chorion) thickness was measured directly on the sections using the software.

### Transmission electron microscopy

Egg cases from several egg batches were cut into 1mm² squares and transferred immediately into a fixative solution (glutaraldehyde 3.2%, carbohydrate buffer 0.31 M, saccharose 0.25 M) for 18 h at 4°C. Then, samples were washed in rinsing solution (0.4 M sodium cacodylate, 0.3 M sucrose) and post-fixed with 1% OsO_4_ in cacodylate buffer 0.2 M, with 0.36 M sucrose (pH 7.4) at 4°C. The egg squares were washed and dehydrated in increasing concentrations of ethanol (70–100%) and progressively included into Epon Embed 812. Protein samples were placed on carbon/formwar coated grids (or carbon formwar grids), fixed with 1% glutaraldehyde for 1 h, and negatively stained with uranyl acetate 1,5% 3 times. Ultrathin sections (80 nm) were prepared and contrasted with 2.5% uranyl acetate followed by lead citrate. Egg capsule sections and protein samples were observed with a 1011 JEOL transmission electron microscope equipped with an Orius 200 GATAN camera and a digital micrograph, and treated by Analysis Five software.

### Scanning electron microscopy

Bacteria were rinsed in cacodylate buffer 0.4 M, pH 7.4, in the presence of 0.3 M sucrose, and fixed with 2.5% glutaraldehyde in cacodylate buffer 0.31 M, pH 7.4, in the presence of 0.25 M sucrose at 4°C for a week. During this fixation period, bacteria were sedimented onto Thermanox coverslips coated with poly-l-lysine for several days. The cells were rinsed 3 times in cacodylate buffer 0.4 M, pH 7.4, in the presence of 0.3 M sucrose. Bacteria were then dehydrated in progressive baths of ethanol (70–100%) and critical point dried (CPD 030 LEICA Microsystem). The cells were sputtered with platinum and observed with a JEOL 6400F scanning electron microscope.

### Protein extraction

Egg protein extraction from several egg batches was performed with four different buffers at two temperatures. Ten outer egg layers were ground in liquid nitrogen and homogenized in 0.1 M Citrate-Phosphate alone, with 100 mM DTT, 5% SDS, or both. Half of the extract was shaken for 1h at room temperature, and the other half was boiled for 10 min. Three main nidamental glands (MNG) from mature female cuttlefish were ground in liquid nitrogen, homogenized in Citrate-Phosphate buffer (0.1 M citrate-phosphate, 5% SDS, 100 mM DTT) with a 1:10 w/v ratio, and boiled for 10 min. All extracts were centrifuged at 40 000 x g for 10 min, and supernatants were recovered. Protein concentrations were determined using Bradford’s method [39].

### Gel electrophoresis

Protein extracts (40 μg) were separated on SDS-polyacrylamide gels (Tris–HCl 4–10%) for 2 h at 120 V in a Tris–HCl migration buffer (25 mM Tris–HCl pH 8.3, 192 mM glycine, 0.1% SDS) with a ColorBurst Electrophoresis Marker (Sigma-Aldrich). Then, the gels were stained with a methanol/acetic acid solution containing 0.1% Coomassie Blue G250 or with PAS.

### PAS staining on polyacrylamide gel

Glycosylated proteins were stained on SDS-PAGE with PAS as described in [6,40]. After migration, the 10%-polyacrylamide gels were incubated in Triton X-100 2.5% for 40 min at 20°C to remove the SDS. The gels were washed in distilled water for 1 h under slow agitation. Then, the gels were immersed in a 7.5% acetic acid solution at 20°C for 1 h. Next, they were soaked in a 0.1% periodic acid solution at 20°C for 45 min, washed quickly in distilled water and immediately incubated in Schiff’s staining reagent (Sigma-Aldrich) in the dark at 4°C overnight. The gels were destained in the dark at 20°C in a 10 mM HCl solution containing 0.1% sodium metabisulfite.

### Protein Identification

All MNG bands from egg protein extracts were excised from the SDS-polyacrylamide gels, reduced with 100 mM DTT, alkylated with 50 mM iodoacetamide, and hydrolyzed with trypsin at 25 ng/μL. The resulting tryptic peptides were recovered from the gels and analyzed by mass spectrometry with the same parameters as described in Cornet *et al*. [41]. Fragmentation patterns were used to determine peptide sequences. Database searching was performed using the Mascot 2.2.04 program (Matrix Science) based on a homemade *Sepia officinalis* database.

### Tissue-specific expression of SepECP transcripts

Total RNAs from five female and five male genital apparatuses and central nervous systems were separately extracted in TriReagent and reverse-transcribed using MMLV-Reverse transcriptase (Promega) according to the manufacturer’s protocol. Amplification reactions were performed on a Biorad Thermocycler under the following conditions: 95°C for 5 min, 30 cycles (95°C for 45 s; 55°C for 45 s; 72°C for 1 min) and a final elongation time of 10 min at 72°C. Reaction mixes were composed of 2μL of cDNA, 1U of Go Taq (Promega) and 10 mM of specific primer for each SepECP chosen in the specific regions (Table 1). The target-specific amplified products had expected sizes of 1,820 and 1,056 bp for SepECP1 and SepECP2, respectively, 261 bp for actin and 406 bp for the elongation factor EFγ. Amplification products were verified by electrophoresis migration on agarose gels, and cDNA sequencing was performed at the Genomic Platform in Nantes (France).

**Table 1:** Oligonucleotide sequences.

Name	Sequences	Position (Nt^a^)
SepECP1	5'-GGAGGCACTTGTACACCGCAAAAGG-3'	104–129
SepECP1Rev	5'-TCTGCAGCAAATTGGCCTACGCC-3'	1660–1683
SepECP2	5'-TCGCTGCTGTTCCTCAGCACTTTGG-3'	22–47
SepECP2Rev	5'-TTTGAGGCCTCTTGGGCAGCCCT-3'	1739–1762
Actin	5’-TCCATCATGAAGTGCGATGT-3’	37–56
ActinRev	5’-TGGACCGGACTCGTCATATT-3’	278–297
EFγ	5’-TACAGCGGGGCAAACGTGACTG-3’	70–91
EFγRev	5’-GGGTGATACGTTCACCCACCAGA-3’	453–475

^a^Nucleotide.

In addition, large-scale Illumina sequencing of different *Sepia officinalis* organs yielded 16 transcriptomes. More specifically, total RNAs from different organs of the genital apparatus or central nervous system of five mature females were extracted separately in Tri-Reagent (Sigma). For each organ, a library was prepared using TruSeq RNA library Preparation Kit v2 (Illumina, Part# 15008136 Rev. A) as already described by Cornet *et al*. [41].

### Bioinformatic analysis

Sequence screening and assembling, transcript annotation were performed with Trinotate software (http://trinotate.sourceforge.net/). Secretory signal peptide sequence prediction was performed using the online software SignalP 4.1 (http://www.cbs.dtu.dk/services/SignalP/) [42], molecular weights (Mw) and isoelectric points (pI) were calculated using Expasy compute pI/Mw tool (http://web.expasy.org/compute_pi/). N-glycosylation sites were predicted using NetNGlyc 1.0Server (http://www.cbs.dtu.dk/services/NetNGlyc/). Sequence alignment was performed using CLC Main Workbench6 (http://www.clcbio.com/). The software Tablet 1.14.10.20 [43] was used to verify transcript sequence coverage. The abundance of egg case protein transcripts in the main and accessory nidamental glands, the oviductal gland and the central nervous system was confirmed by the number of Fragments Per Kilobase of exons per million fragments Mapped (FPKM) from the whole Sepia transcriptome assembly.

### Bacterial challenge on eggs

Eggs about to hatch (80 days) were collected and were either washed three times in 0.2 µm-filtered seawater or incubated in sterilized seawater with 3% ampicillin / 1% gentamycin for 1h at 18°C and washed three times in seawater before being used. Eggs were incubated 48 h in infected water containing the cuttlefish pathogen *Vibrio alginolyticus* [44] in the exponential growth phase (0.2 units of optical density at 600 nm (OD)). Bacterial growth was measured after incubation (OD1). Then, bacteria were recovered by filtering the seawater on 0.2 μm and incubated in Zobell medium overnight at 18°C. Then the OD was read at 595 nm (OD2). Three controls were used: (i) *Vibrio alginolyticus* growth in seawater at 18°C for 48h, (ii) eggs with or without antibiotic treatment to inhibit possible endogenous bacterial growth, and (iii) sterilized seawater only. Statistical analysis was performed using Student’s test to test for differences between the *Vibrio alginolyticus* growth control and the two following samples: “eggs exposed to *Vibrio alginolyticus*”, or “eggs exposed to *Vibrio alginolyticus* and treated with 3% ampicillin / 1% gentamycin”.

### Protein antibacterial activity

The antimicrobial activity of egg case protein extracts was assayed on strains of the Gram-positive bacterium *Bacillus megaterium*, and on the Gram-negative bacteria *Escherichia coli*, *Vibrio splendidus*, and *Vibrio alginolyticus* used by Duval and collaborators [45]. Egg-case protein extracts were first precipitated to eliminate SDS and DTT from the citrate-phosphate buffer. Briefly, four volumes of acidified (0.1N HCl) acetone/methanol (v:v) solution were added to one volume of protein extract and kept at -20°C for one hour. Each protein precipitate was then centrifuged at 4°C, 10 min, 12 000 x g, the supernatant was discarded, and the protein pellet was dried and dissolved in water. Antimicrobial assays were performed in 96-well plates in technical triplicate for each sample according to Bulet *et al*. [46]. Each well contained ten microliters of protein extract dilutions, ampicillin or seawater with 100 μL of bacterial culture diluted at a starting optical density of OD_595nm_ = 0.001 in poor Zobell medium (peptone 4g/L, instant ocean salt 30 g/L) for *Vibrio splendidus*, *Vibrio aestuarianus* and *Vibrio alginolyticus*, and in poor broth (10 g/L bacto-tryptone, 10 g/L NaCl) for *E*. *coli* and *B*. *megaterium*. Microplates were incubated for 16 h at 20°C for vibrios, and 30°C for *E*.*coli* and *B*. *megaterium*. Finally, the OD_595nm_ was measured, and bacterial growth was determined. The bactericidal activity of the antibacterial fractions was determined on nutrient Zobell or LB agar plates after 16 h of incubation.

## Results

### Structural study or the egg case during embryonic development by photonic microscopy and transmission electron microscopy

**Fig 1:**
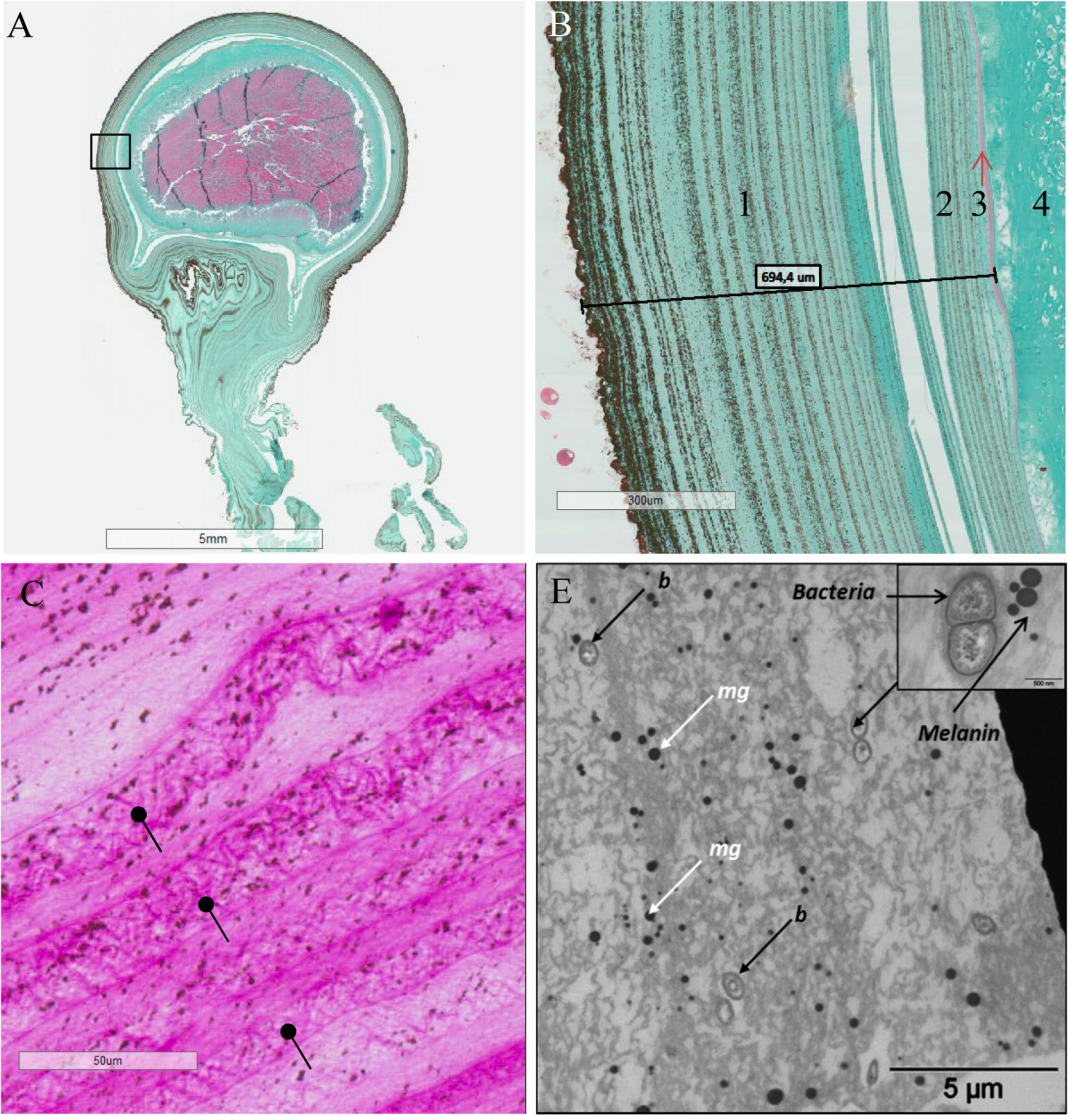
Egg Case and SepECP structure analysis by histological techniques. (A) Longitudinal section of a whole 15-day-old egg. Black box, magnificence zone of Fig 1.B. (B) Longitudinal section of a 15-day-old egg stained in Prenant-Gabe trichromic. 1: outer layer of the extrachorion, 2: inner layer of the extrachorion, 3: chorion, 4: vitelline membrane, 5: oocyte. (C) Longitudinal section of a 24-hour-old egg focused on the outer layer of the extrachorion stained in Periodic Acid of Schiff. Glycoproteins polymerize and are assembled in narrow meshes (black arrows) that are organized in layers. (D) Section of the outer layer of the extrachorion of an egg in TEM (b, bacteria; mg, melanin granules).

Structural analysis of the egg capsule by photonic microscopy revealed a lamellar structure of the inner and outer envelopes (Fig 1A and 1B), with successive spirally wound layers. The outer envelope contained melanin granules gathered in layers that became increasingly intense. Sections performed from spawning to hatching underlined a decrease in capsule thickness. One day after egg-laying, the egg capsule was about 1.4 (+/- 0.6 mm) μm thick, and then its thickness decreased throughout embryogenesis. It was about 716.5 (+/- 150 μm) after 15 days (Fig 1B), 437.9 (+/-104 μm after 35 days and finally down to 231.1 (+/- 110) μm just before hatching (data not shown).

PAS staining on sections of 1-day-old eggs showed the presence of polysaccharide components or glycoproteins in the egg case (Fig 1C). Interestingly, the distribution of the staining in the outer layer was not homogeneous and underscored the presence of a dense mesh.

The observation of the outer envelope by TEM showed the presence of melanin deposits and revealed the occurrence of isolated or grouped structures whose size ranged between 0.4 and 1 μm and whose morphology was compatible with bacterial structures (Fig 1D). The egg case ultrastructure showed a narrow mesh composed of glycoproteins and polysaccharides.

### Proteomic analysis of the Egg capsule and Main Nidamental Glands

**Fig 2:**
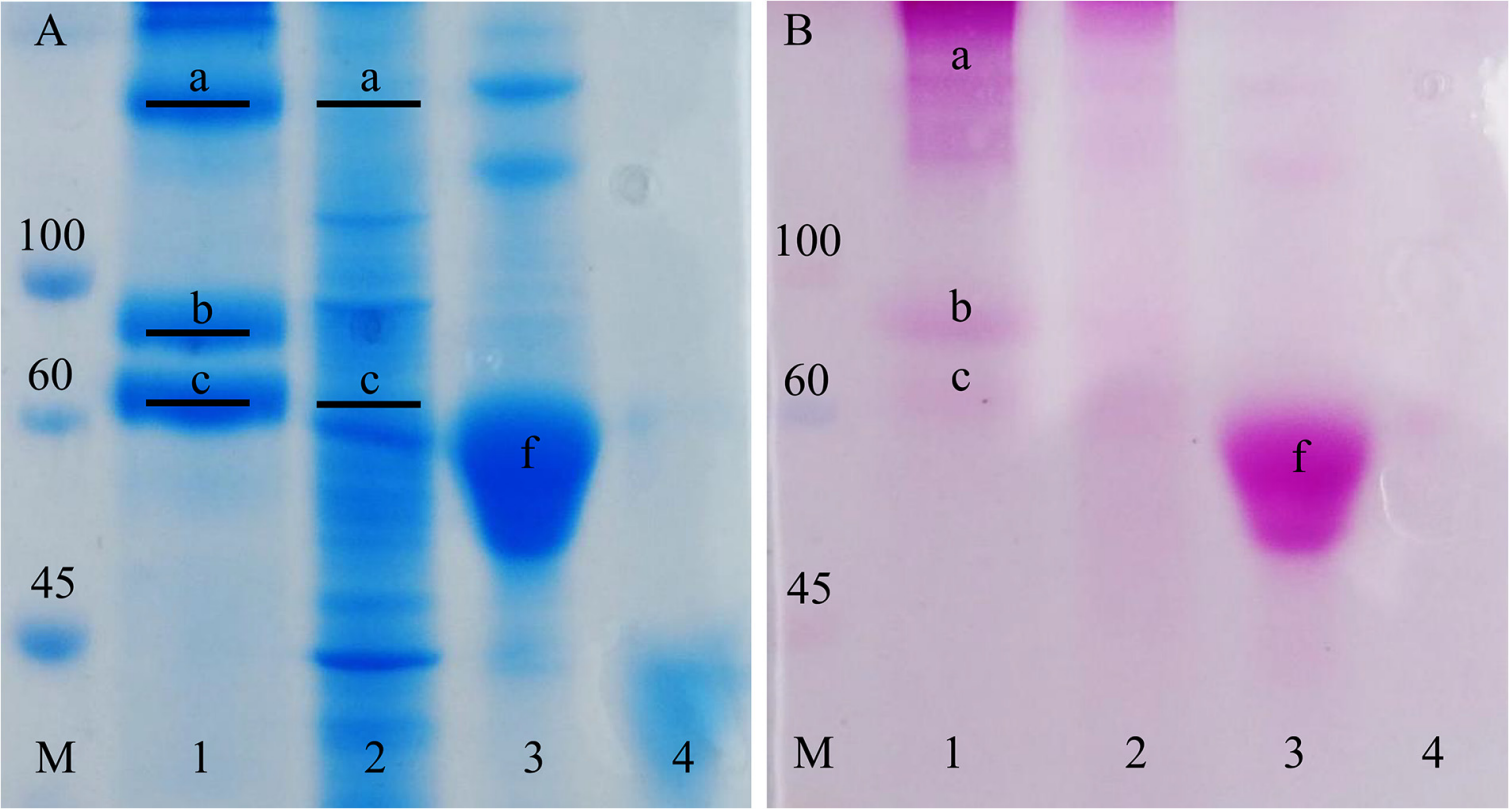
Egg case and Main Nidamental Gland protein and glycoprotein profiles. M: Molecular weight standard, 1: Egg Case protein extract, 2: Main Nidamental Gland protein extract, 3: fetuin, 4: β-lactoglobulin. (A) SDS-PAGE profiles of Egg Case and MNG protein extracts stained in Coomassie blue. a: 135kDa band containing SepECP 1 and SepECP 2, b: 75kDa band containing SepECP 2, c: 60kDa band containing SepECP 1. Black lines show bands submitted to tryptic digestion and MS/MS identification. (B) SDS-PAGE stained in PAS. a: 135kDa band containing SepECP 1 and SepECP 2, b: 75kDa band containing SepECP 2, c: 60kDa band containing SepECP 1, f: fetuin.

**Fig 3:**
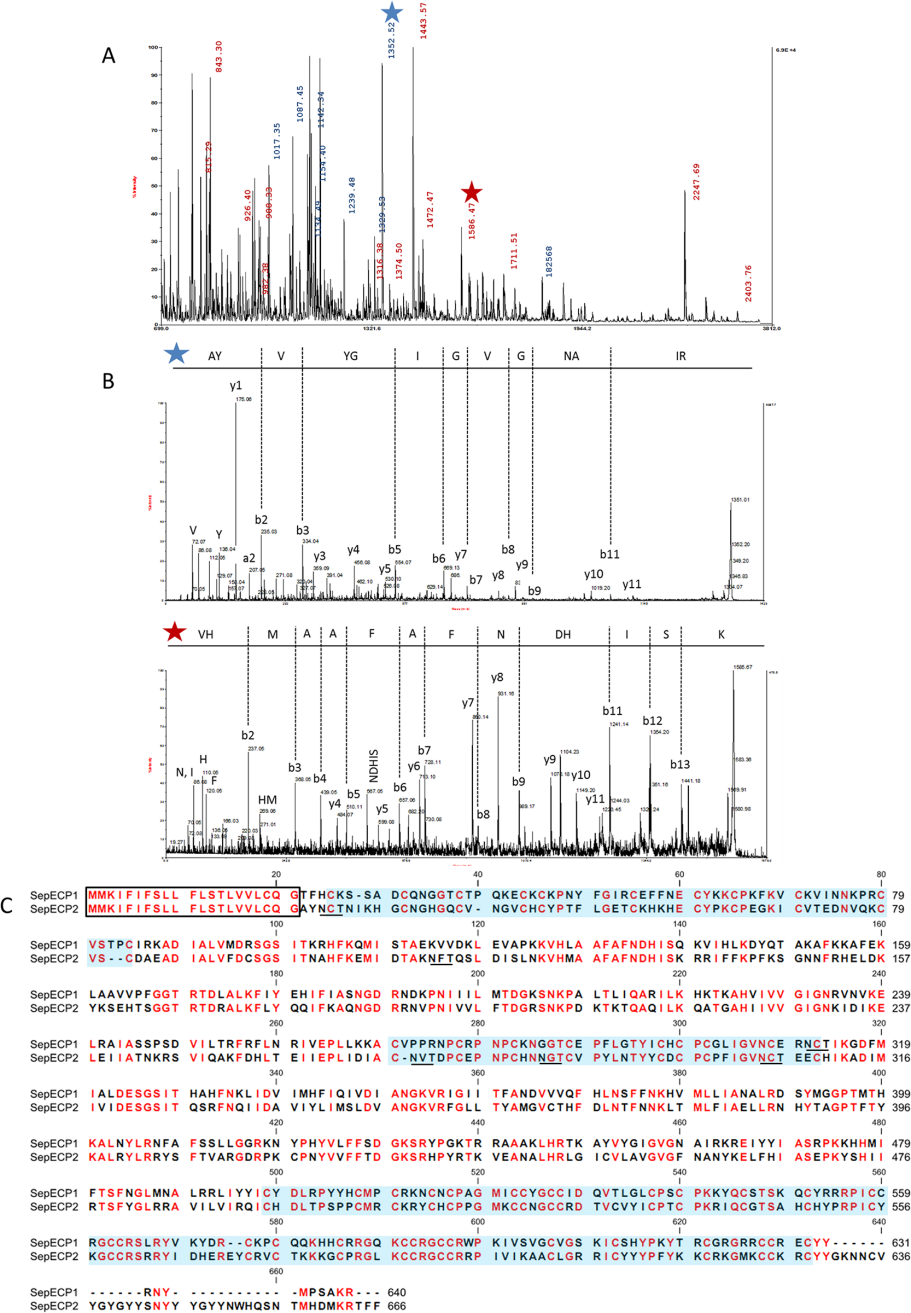
Identification of Egg Case proteins in the Main Nidamental Gland (MNG) and egg case (EC). (A) MS spectrum of the tryptic peptide from the 135kDa band. In blue, m/z corresponding to SepECP1, in red, m/z corresponding to SepECP2. (B) MS/MS spectra of the two tryptic peptides AYVYGIGVGNAIR (m/z = 1352.52) and VHMAAFAFNDHISK (m/z = 1586.47) recovered from SepECP 1 and SepECP 2 proteins, respectively. (C) SepECP sequence alignment. In red, conserved amino acids; black frame, signal peptide; N-glycosylation predicted sites are underscored; blue box, cysteine-rich conserved domain.

The protein profile of *Sepia officinalis* main nidamental glands and egg capsule was established by coupling SDS-PAGE with MS/MS analysis. Citrate-phosphate buffer containing a high level of detergent (SDS) with or without a reducing agent (DTT) and boiled for 10 min exhibited the best protein yield (data not show). The most stringent protocol was chosen for its ability to dissolve and solubilize egg case proteins. Egg case electrophoretic profiles yielded molecular weights of ~60–75 kDa and ~140k Da (Fig 2A). After digestion treatment, tryptic peptides were analyzed by MS. Two unknown proteins were identified in the egg case protein extract using MASCOT from the whole sepia transcriptome with no available annotation in the NCBI database. The first one was found in the lower band at ~60 kDa and the second one was identified in the band at ~75 kDa. The upper band contained a dimer of these two proteins made of respectively 12 and 11 unique tryptic peptides, with MASCOT scores of 576.32 and 542.87. The MS spectrum highlighted a high number of identified peptides from these two proteins apparently present in the same proportion in the band at 135 kDa (Fig 3A). We detailed one MS/MS tryptic peptide spectrum from each protein (Fig 3B). MS/MS profiles were validated manually. Coverage rates were 42.66% for SepECP 1, and 34.93% for SepECP 2 (Fig 3D). These newly identified proteins, with respective calculated masses of 71 kDa and 74 kDa, were named SepECP 1 and 2, for *Sep*ia *officinalis* Egg Case Proteins. Corresponding MNG bands confirmed the identification of SepECP1 (MASCOT score: 248.34) and of the dimer complex SepECP1 (MASCOT score: 273.90) and SepECP2 (MASCOT score: 47.87).

**Fig 4:**
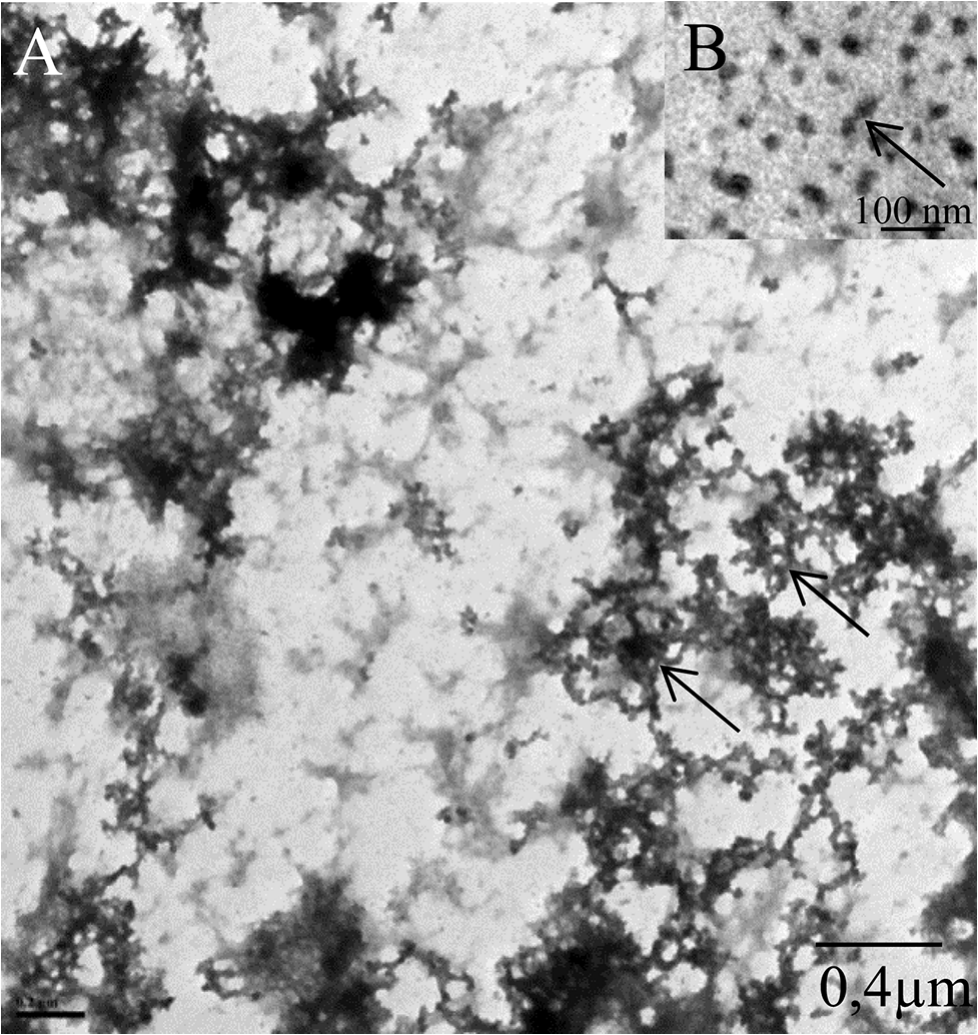
SepECPs forming a mesh observed in TEM (A) SepECP protein extract precipitated and solubilized in sterile seawater observed in TEM. Black arrow, protein network. (B) SepECP protein extract precipitated, solubilized and boiled in sterile water observed in TEM. Red arrow, protein alone.

When SepECP1 and SepECP2 were aligned (Fig 3C), they turned out to possess an identical signal peptide. They displayed 49% homology, with 3 highly conserved cysteine domains. SepECP1 and SepECP2 contained 50 and 55 cysteines, respectively, therefore they can be considered as cysteine-rich proteins. These cysteines could be implied in intramolecular and intermolecular disulfide bonds involved in the formation of heterodimers evidenced by SDS-PAGE. Observation of SepECPs by TEM indicated that these proteins gather in strings that form a network similar to the network observed in egg case TEM sections (Fig 4A). Interestingly, boiling the protein extract broke down the string structure, and highlighted the presence of SepECPs in isolated globular structures (Fig 4B). Moreover, the conserved motif CX_2_GX_2_CCXGCCX_8-9_C occurring in the third cysteine-rich domain is repeated twice in both protein sequences. The prediction of N-glycosylation sites indicated that these could be glycosylated proteins as there are respectively 1 and 5 N-glycosylation sites in SepECP1 and SepECP2. PAS staining of SDS-PAGE gels confirmed the glycosylation prediction of both SepECPs (Fig 2B). PAS staining intensity was stronger in SepECP 2 bands than in SepECP 1 bands, suggesting a higher level of glycosylation of SepECP 2. SepECPs 1 and 2 are cationic proteins that are rich in basic amino acids (lysine and arginine: 17.2% and 14.3%) and poor in acidic amino acids (glutamic and aspartic acid: 6% and 7.7%), hence their positive charge (+70 and +42).

### Tissue-specific expression pattern of SepECP transcripts

**Fig 5:**
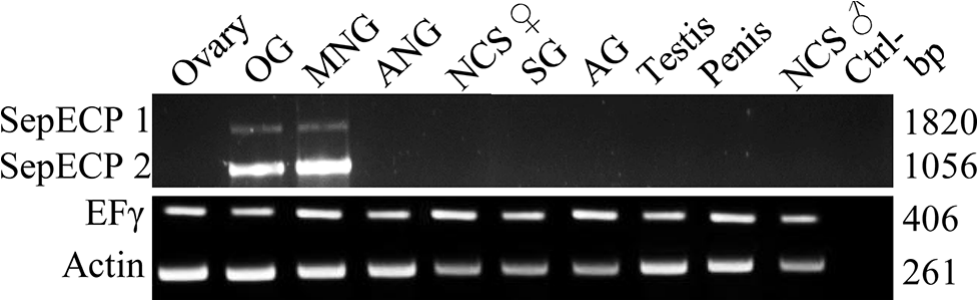
Tissue-specific expression patterns for SepECPs by RT-PCR. Expression patterns for SepECP 1, SepECP 2, elongation factor EFγ and actin transcripts in Ovary; OG, Oviductal Gland; MNG, Main Nidamental Gland; ANG, Accessory Nidamental Gland; CNS, Central Nervous System; SG, seminal Gland; AG, Accessory Gland. Ctrl−, negative control (no template added to the PCR mix).

SepECP transcript expression specificity was assayed by RT-PCR with specific primers designed in the variable regions of SepECP 1 and 2, from mRNAs from five male and five female genital apparatuses and central nervous systems (CNSs) (Fig 5). We observed amplification products only in the main nidamental gland (MNG) and oviductal gland (OG) samples. SepECP transcripts were not detected in male genital apparatus. We used *Sepia officinalis* Illumina transcriptomes to verify SepECP transcript expression levels in female genital apparatuses and CNS (Table 2). SepECP transcripts were exclusively found in two female genital organs: MNG and OG. However, they were mainly expressed in the main nidamental glands, where they represented the two most expressed transcripts, with 126 145 and 118 536 FPKM in MNG for SepECP 1 and SepECP 2 transcripts, respectively. This was four times more than in OG, with 29 844 and 33 367 FPKM, respectively.

**Table 2:** Tissue-specific expression patterns of SepECPs using Illumina sequencing.

	Illumina transcripts (FPKM^g^)				
Sequence	Female genital glands				
Name	Ov^b^	MNG^c^	ANG^d^	OG^e^	CNS^f^
SepECP1	0	126 145	17	29 844	0
SepECP2	1	118 536	13	33 367	0
Efα^a^	1 527	1 134	1 678	1 765	1 860

Transcript expression of SepECP 1, SepECP 2 and

^a^Elongation Factor in

^b^Ovary

^c^Main Nidamental Gland

^d^Accessory Nidamental Gland

^e^Oviductal Gland and

^f^ Central Nervous System.

^g^Fragments per kilobase of exons per million fragments mapped.

**Fig 6:**
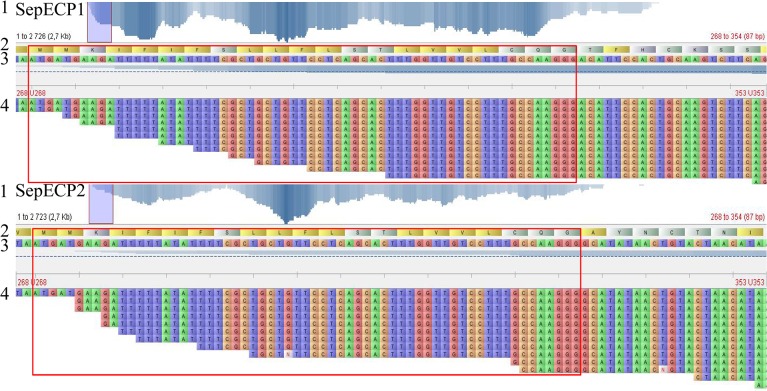
SepECP transcript coverage obtained from Main Nidamental Gland transcriptome. (1) In SepECP 1 and SepECP 2, respectively: Bases with coverage: 76.119% and 90.158%; Average coverage rate: 9.198 and 10.959; Maximum coverage depth: 22 and 34 reads. (2) N-terminal SepECP protein sequence, red frame: signal peptide. (3) 5’-end transcript sequence. (4) 5’-end read coverage.

Sequence checking using Tablet software indicated that both signal peptides had the same nucleotide sequence, with good read coverage (Fig 6). The signal peptides were covered with up to 12 reads for SepECP 1 and 9 reads for SepECP 2. Moreover, SepECP nucleotide sequences were different directly after the last nucleotide of the signal peptide, and read average size was around 250 bp, assembly mistakes on the signal peptide excluded.

Sequences are available from Genbank under Bioproject PRJNA242869 and Biosample accession No: SAMN02709769.

### Antimicrobial assays

The bacterial challenge performed with the eggs treated (OD1: 0.006 uOD) or not with antibiotics (OD1: 0.007 uOD) on *V*. *alginolyticus* showed significant growth inhibition compared to *V*. *alginolyticus* alone (OD1: 0.042 uOD) When eggs were challenged with *V*. *alginolyticus* bacteria, with or without antibiotic treatment, significant growth inhibition compared to *V*. *alginolyticus* alone was evidenced (Table 3). Incubating eggs without *V*.*alginolyticus* confirmed the presence of living bacteria inside the egg case. Treatment with 3% ampicillin/ 1% gentamycin was inefficient on egg-harbored bacteria: growth was observed in Zobell medium after overnight incubation.

**Table 3:** *Vibrio alginolyticus* infection of *Sepia* eggs about to hatch, previously treated or not with antibiotics.

	Eggs		Eggs with A/G^a^	
Sample	OD1 SWb	OD2 Zb^c^	OD1 SW^b^	OD2 Zb^c^
Eggs	0,003	>1	0,002	>1
Eggs + *Vibrios alginolyticus*	0,006 ***	>1	0,007 ***	>1
*Vibrio alginolyticus*	0,042	>1	0,042	>1
Filtered seawater	0	0	0	0

^a^Eggs with Ampicillin/Gentamycin.

^b^Optical Density in seawater.

^c^Optical Density in Zobell medium.

***, p ≤ 0.001.

We tested the activity spectrum of the EC protein extract on three marine bacterial strains of *Vibrios* and two human pathogens, *E*. *coli* and *B*. *megaterium* (Table 4). EC extract containing only SepECP proteins exhibited antibacterial activity against *V*. *alginolyticus*, *V*. *splendidus*, *V*. *aestuarianus* and *E*. *Coli* at concentrations ranging between 10 and 200 μg/mL according to the bacterial strain. The extract was only bactericidal on *V*. *alginolyticus* at 100μg/mL. SepECP was inefficient against the GRAM+ strain *B*. *megaterium*.

**Table 4:** Inhibitory and bactericidal activity of SepECP proteins.

		Inhibitory activity			Bactericidal activity
Bacteria	Strains	EC extract	Ampicillin	Gentamycin	EC extract
		(μg/ml)	(μg/ml)	(μg/ml)	(μg/ml)
GRAM +	*Bacillus megaterium*	NA	oct-20	0.1–1	NA^a^
	*Escherichia coli*	10	1–2.5	0.1–1	NA^a^
GRAM -	*Vibrio splendidus*	100–200	05-oct	0.1–1	NA^a^
	*Vibrio aestuarianus*	<10	1–2.5	2.5–5	NA^a^
	*Vibrio alginolyticus*	10–100	>320	2.5–5	100

^a^No Activity.

### *Vibrio aestuarianus*/SepECPs complex

**Fig 7:**
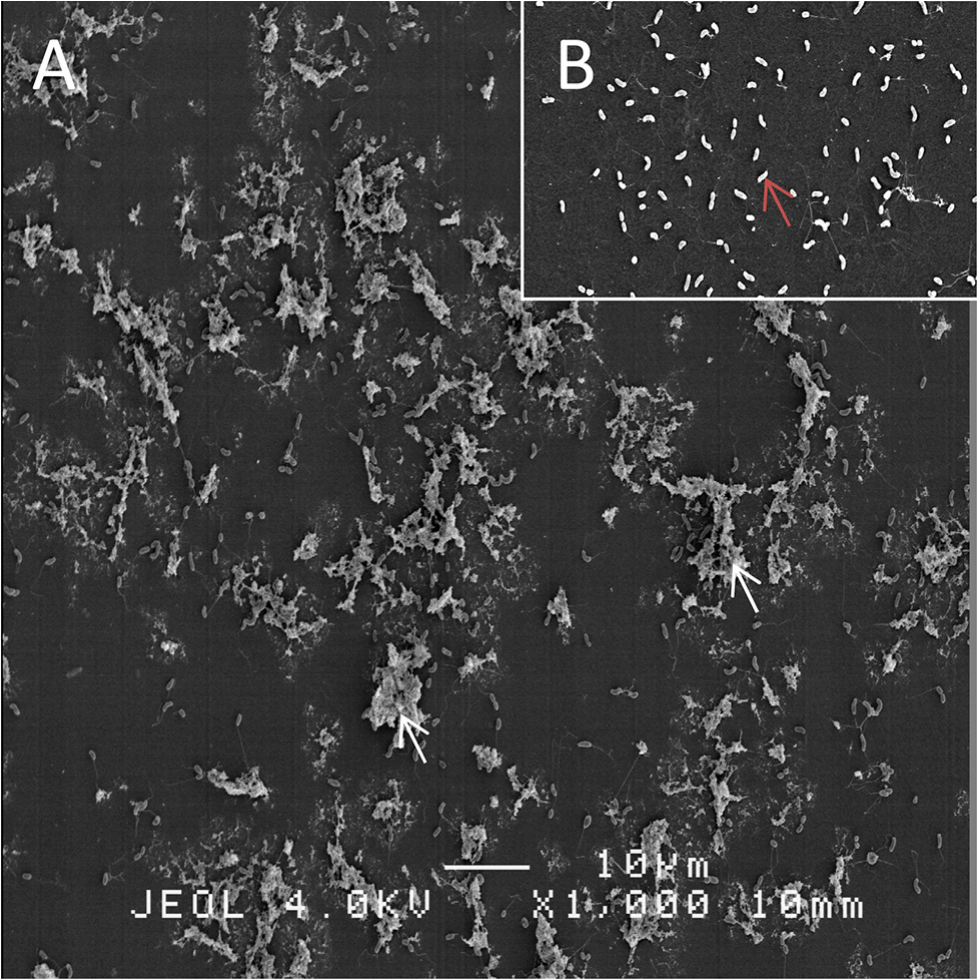
SepECP/*Vibrio aestuarianus* complex in SEM. (A) *Vibrio aestuarianus* with SepECP agglomerates (white arrows). (B) *Vibrio aestuarianus* without SepECPs (red arrow).

SEM observation of the bacterium *V*. *aestuarianus* in contact with SepECP proteins used for antimicrobial assays revealed the formation of many agglomerates (Fig 7A), with a few bacteria glued and trapped inside a protein complex. No bacterial agglomerate was observed in the absence of SepECP proteins (Fig 7B).

## Discussion

The egg case is essential in the reproductive strategy of the cuttlefish *Sepia officinalis* [30]. This capsule represents the only barrier against environmental aggressions, so it undergoes huge changes that are in accordance with embryo protection and development [31]. Our study focuses on the characterization and the function of proteins composing the outer egg case. We identified a new protein family composed of two proteins called SepECP 1 and SepECP 2, secreted by the main nidamental glands and sharing high sequence homology. Proteomic analysis revealed that these proteins constitute the main protein component of the egg capsule.

Studying egg case matrix proteins remains tricky. For example, in dogfish egg case, collagenous proteins proved very resistant to mechanical and enzymatic disruption thanks to extensive covalent cross-linking [6]. For *Sepia* egg case proteins, the use of high quantities of denaturing and reducing agents to dissolve and break protein bonds was essential. SepECPs had no available annotation in the NCBI database, except some cartilage matrix proteins that shared less than 30% identity. The high cysteine rate of SepECPs rules out structure homologies with collagen-like proteins identified in mollusk, fish or avian egg cases [11,21,47]. The capsulin found in the egg capsule of the gasteropod *Aplysia* is twice the size of SepECP and contains fewer cysteines distributed along the whole sequence [25]. The high rate of cysteines in SepECP sequences could allow us to classify these proteins in the cysteine-rich protein family already described in several avian, reptile and insect eggs [48,49]. Structural proteins with high cysteine contents have indeed been found in the eggshell of the silkmoth *Bombyx mori* [48]. Moreover, Cysteine Rich Eggshell Membrane Proteins (CREMPs) from bird and reptile eggs contain similar cysteine patterns to SepECPs’. For example, hen CREMP contains a repeated C-X_4_-C-X_5_-C-X_8_-C-X_6-11_ pattern that is involved in fibers forming layers over the egg white prior to deposition of the mineralized shell [49]. However, unlike CREMPs, SepECPs contain only few cysteine-rich patterns distributed in the three conserved domains of the two proteins. *Sepia* egg case proteins appear to be closer to clusterin, a matrix eggshell glycoprotein that contains two cysteine-rich domains [50]. The protein found in the outer layer of the eggshell is cleaved into two α/β subunits of 35 kDa linked by di-sulfide bonds. In addition to a structural role, this protein could bind and stabilize slowly aggregating proteins. The heterodimeric configuration highlighted by proteomic analysis is consistent with the configuration already observed in hen eggshell. Yet, these cysteine-rich domains could be involved in the intramolecular and intermolecular bonds that allow the formation of the narrow mesh observed in TEM. However, according to the high level of lysine residues in SepECPs, protein bonds could also involve lysine cross-links already described in structural proteins [51,52].

Egg case optical microscopy observations highlighted the formation of an organized network comparable to the one described in dogfish egg case [19]. Observation of the SepECP complex in SEM suggests that SepECP proteins are indeed directly involved in egg case matrix formation. The narrow mesh formed by the protein/mucopolysaccharide complex is an efficient polymer with elasticity and resistance properties that confers resistance to mechanical aggressions and bacterial penetration [53]. Our histological study of *Sepia officinalis* egg case showed a lamellar structure composed of muco-substances, i.e. polysaccharides already observed in opisthobranchia [54], melanin, and bacteria probably maintained together by the SepECP complex. Interestingly, the ink produced by cephalopods contains melanin, and is only incorporated into eggs of some sepioidea species [30]. Pigment incorporation into hen eggs may protect them against predators as the egg is less conspicuous [55]. Moreover, ink from cephalopods affects the consummatory and ingestive phases of predation of both sea catfish and summer flounder [56]. Therefore, incorporation of ink and melanin into *Sepia* eggs could protect the embryo against predation.

The bacteria found inside the egg case were previously identified in cephalopod eggs [57,58]. In *Loligo opalescens* eggs, these bacteria are thought to be involved in embryo defense by avoiding other bacterial colonization or by producing antimicrobial substances [59]. The symbiotic bacterium genus *Alteromonas* present in the eggs of the shrimp *Palaemon macrodactylus* produces a fungicide substance against the fungus *Lagenidium callinectes* [60]. In addition to this potential chemical protection by ink and bacteria, egg case proteins provide antimicrobial protection. Benkendorff and collaborators suggested that physical protection by this natural barrier could be insufficient and may be supplemented by chemical defense. They demonstrated that both gelatinous egg mass and egg capsule from 39 mollusks inhibited microbial growth [28]. The bacterial challenge with *Vibrio alginolyticus* on *Sepia* eggs underscored the eggs’ antimicrobial activity. Young egg masses from the nematode *Meloidogyne javanica* induced strong agglutination and reduced growth rates of both *Bacillus subtilis* and *Saccharomyces sp*. [61].

*Sepia* eggs also appear to have antifouling properties as no bacterial biofilm was observed during the bacterial challenge or embryo-developing period. The egg mass extracts of the marine gastropods *Chicoreus virgineus*, *Chicoreus ramosus* and *Rapana rapiformis* possess wide-spectrum antimicrofouling activity against 40 biofilm bacteria [24]. Our SEM observations demonstrated that SepECPs were able to agglomerate GRAM- bacteria like *Vibrio aestuarianus*. Thus, the bacteria bound to SepECPs cannot penetrate the egg case to infect the embryos and are unable to divide. Furthermore, the egg case protein extract containing quite exclusively SepECP 1 and 2 exhibited efficient antimicrobial activity on GRAM- bacteria, with strong activity on three *vibrio* strains and a strong growth-inhibiting effect on *E*.*coli*. Moreover, SepECP activity was resistant to denaturation and heating. Egg wax proteins from the African cattle tick *Amblyomma hebraeum* contain heat-stable antimicrobial proteins resistant to proteinase K [62].

Despite some homologies to other proteins in size or glycosylation, the structure of SepECPs remains atypical. First, this egg protein family is original due to the presence of a unique signal peptide for both proteins. It suggests that a unique gene encodes both SepECP transcripts by alternative splicing. Among all egg case proteins, no evidence of an identical signal peptide for several proteins has ever been found before. However, this originality was already observed in mollusk neuropeptide precursors encoded by a unique gene, like FaRPs or Luqin [63–65].

*Sepia* egg case proteins are highly cationic proteins containing an unusual basic amino acid composition (more than 15%) and very few acidic amino acids, hence a high positive charge. Futhermore, SepECP proteins are glycosylated judging from N-glycosylation prediction, and PAS staining indicated the involvement of glycoproteins in the formation of an organized network. In *Aplysia kurodai* eggs, chemical defense is ensured by high-molecular-weight glycoproteins Aplysianin A (320 kDa) and E (250 kDa) [26,27,66,67] or glycoproteins Julianin E and G that display closer molecular weight values to SepECPs [68]. These glycoproteins exhibited bacteriostatic, antifungal and antitumor activities, and are resistant to proteolytic enzyme treatments. However, these proteins do not seem to be involved in egg matrix proteins like SepECPs. Still, hen eggshell matrix proteins have been described for their role in natural antimicrobial defense [16,69]. *Pseudomonas aureginosa*, *Bacillus cereus and Staphylococcus aureus* were inhibited in the presence of soluble eggshell matrix proteins (100 μg/mL) in the same concentration range as SepECP on *Vibrios* [16]. Moreover, the egg membrane protein Ovocalyxin-36 exhibited lipopolysaccharide- (LPS-) binding activity and bound LPSs from *Escherichia coli*. Interestingly, this protein shares sequence homology with bactericidal permeability-increasing proteins (BPIs), lipopolysaccharide-binding proteins (LBPs) and palate, lung and nasal epithelium clone (PLUNC) proteins [17].

The newly identified proteins do not exhibit such homologies with immune effectors Based on (i) the absence of repeated sequences along the protein sequences, (ii) the cationic properties conferred by the high rate of basic amino acids, and (iii) the three original conserved cysteine-rich domains, SepECP proteins appear to belong to a new class of proteins. In the first week of embryo development, SepECP 1 and SepECP 2 form a narrow mesh constituting a natural barrier against environmental aggressions and exhibit antimicrobial activity against aggression by *Vibrios*.

During embryo development, the egg case becomes increasingly thin, while retaining elasticity to allow for embryonic growth. The initially sealed capsule gradually lets seawater in, while its outer layers are slowly lost by delamination. SepECPs are probably cleaved to allow for hatching at the end of embryo development. The small cleaved proteins and peptides could be involved in water entry into the vitellin cavity, as suggested by Gomi *et al*. [70], and could also provide immune defense according to the protein cationic profiles.
